# Resident T cell activation leads to human hair follicle immune privilege loss *ex vivo*, which is prevented by the DHODH inhibitor farudodstat: relevance for alopecia areata

**DOI:** 10.3389/fmed.2026.1810644

**Published:** 2026-04-29

**Authors:** Ilaria Piccini, Thomas Rouillé, Wolfgang Funk, Francisco Jimenez, Alexandre Kaoukhov, Carl Firth, Amos Gilhar, Janin Edelkamp, Ferda Cevikbas, Marta Bertolini

**Affiliations:** 1 QIMA Life Sciences, QIMA Monasterium GmbH, Münster, Germany; 2 Clinic for Plastic, Aesthetic and Reconstructive Surgery Dr. Dr. med. Funk, Munich, Germany; 3 Mediteknia Clinic, Las Palmas de Gran Canaria, Spain; 4 Ciencias de La Salud, University Fernando Pessoa Canarias, Las Palmas de Gran Canaria, Spain; 5 ASLAN Pharmaceuticals, San Mateo, CA, United States; 6 Technion–Israel Institute of Technology, Haifa, Israel

**Keywords:** alopecia areata, DHODH inhibition, farudodstat, hair follicle organ culture, immune privilege, T cell proliferation, T cell receptor

## Abstract

The autoimmune hair loss disorder alopecia areata (AA), is characterized by immune privilege (IP) collapse of the hair follicle (HF) bulb resulting from a Th1-dependent inflammatory response. Although CD8^+^ T cells are recognized key drivers of the disease, it remains to be clarified whether the activation of HF resident T cells suffice to initiate IP loss and thus elicit the cascade of events leading to AA. Here, we utilized the human microdissected HF organ culture model to answer this question by activating intra- and peri-follicular HF resident T cells with αCD3/αCD28 antibodies. TCR stimulation indeed resulted in enhanced resident T cell proliferation, as indicated by significantly increased CD3^+^Ki-67^+^ cells, and higher intrafollicular CD3^+^ T cell numbers. Furthermore, αCD3/αCD28 stimulation promoted key signs of HF IP collapse, by increasing bulbar MHC class I and II expression and elevating MHC class II^+^ cells numbers. We next sought to investigate whether T cell proliferation plays a central role in the TCR activation–dependent collapse of the bulb IP. To test this, we co-administered the DHODH inhibitor farudodstat 1 day prior and during the stimulation with αCD3/αCD28 in HF organ culture. Short-term treatment with farudodstat reduced the increase in T cell proliferation and significantly decreased the upregulated MHC class I and II expression induced by TCR stimulation with αCD3/αCD28. Our results show that stimulation of HF resident T cells via TCR engagement induces an AA–like phenotype in healthy human HFs *ex vivo*, characterized by T cell proliferation and subsequent IP collapse. DHODH inhibition with farudodstat only partially reduces T cell numbers but prevented HF IP collapse induction.

## 1 Introduction

Alopecia areata (AA) is an (auto-)inflammatory disorder affecting the hair follicle (HF) (1, 2), in which immune privilege (IP) collapse and bulbar inflammation result in premature catagen development and/or HF dystrophy, ultimately leading to hair loss (1–3). Regardless of the initial causal event, which may involve either antigen-independent or classical, autoimmune-dependent mechanisms (1), T cell activation and HF (auto-)antigen recognition lie in the center of its pathogenesis (1–3). Yet, it remains to be clarified whether the activation of human HF resident immune cells is sufficient to induce HF-IP collapse and thus elicit the cascade of events leading to AA. Here we have attempted to clarify this by using the gold standard human healthy HF organ culture model (4, 5) and selectively activating the T cell receptor (TCR) of HF resident T cells using αCD3/αCD28 stimulation (6, 7). Alongside the C3H/HeJ mouse model (8) and the humanized mouse model for AA (9, 10), the healthy human HF organ culture has been pivotal in elucidating the role of key AA pathogenic cytokines, as well as mechanisms involved in AA pathogenesis (11–15). Indeed, stimulation of human HFs *ex vivo* with IFNγ results in bulb IP collapse (11–15). Moreover, loss of IP and/or induction of an AA-like phenotype were also recapitulated in this model by mimicking several antigen-independent initiating mechanisms, e.g., by inducing neurogenic inflammation through the administration of substance P (16), or by co-culturing HFs with skin-isolated γδT-cells (15), or blood-generated ILCs (17). In AA, activation of the TCR triggers T cell proliferation, followed by cytokine production. The enzyme dihydroorotate dehydrogenase (DHODH) is essential for the *de novo* synthesis of pyrimidines, a process critical for effective cell proliferation. DHODH inhibition disrupts pyrimidine production and consequently reduces T cell proliferation (18). Several FDA-approved DHODH inhibitors preferentially targeting T cells are currently used to treat (auto-)immune disorders, such as multiple sclerosis (19–22). The novel, orally active and highly selective DHODH inhibitor farudodstat is approximately 30 times more potent (IC50:35 nM) than first-generation DHODH inhibitors and is currently in a Phase 2a proof-of-concept trial for AA (FArudodstat STudy in Alopecia Areata, FAST-AA).

Here, we stimulated the TCR with αCD3/αCD28 in healthy human HFs *ex vivo* to experimentally induce an AA-like phenotype. In a subsequent step, we treated the samples with farudodstat to: (1) assess whether T cell proliferation is necessary for IP collapse, and (2) evaluate whether DHODH inhibition can prevent IP collapse. This approach also allowed us to preclinically assess farudodstat’s potential as a novel therapeutic for AA. Our findings show that TCR stimulation did not affect hair cycle dynamics or hair matrix keratinocyte proliferation. However, it markedly increased T cell proliferation, T cell abundance, and the expression of IP collapse markers MHC class I and II. farudodstat treatment reduced both proliferating T cell numbers and MHC class I/II expression.

## 2 Methods

### 2.1 Donor material and information

Human amputated or full-length HFs were microdissected from healthy scalp skin or follicular units obtained from *n* = 7 healthy donors (22–67 years old), within 24 h after surgical procedure. All human samples have been obtained after informed, written patient consent and ethics committee approval (Monasterium Laboratory Biobank approval 2020-954-f-S, Comité de Bioética de la Universidad Fernando Pessoa Canarias (03 (2020-06-22)). This study was conducted according to the Declaration of Helsinki principles.

### 2.2 Hair follicle organ culture and treatment

Hair follicles were cultured as described before (4, 23) at 37 °C with 5% CO_2_ in WCM plus RPMI [WCM; William’s E minimal media (Gibco, Life Technologies) plus 10 ng/ml hydrocortisone (Sigma Aldrich), 10 μg/ml insulin (Sigma Aldrich) and 1% penicillin/streptomycin mix (Gibco)]. After a resting period of 1 day, anagen VI HFs were randomly allocated to the different experimental groups. For the first part of our study (IP collapse induction) HFs were treated with 25 μg/mL αCD3 and αCD28 antibodies for 4 days *ex vivo*. To test farudodstat as an IP collapse preventing agent, HFs were first treated with 70 nM farudodstat or vehicle control (0.1% DMSO). One day later, on day 2 *ex vivo*, 25 μg/mL αCD3 and αCD28 antibodies were added (BD Pharmingen, clone OKT3 and CD28.2, respectively). After 4 days *ex vivo*, treatments were refreshed and on day 5 cultures were terminated. HFs were embedded in Cryomatrix (OCT) and snap frozen in liquid nitrogen. 6 μm thickness sections were cut with a Leica cryostat, consecutive sections of each HF were collected, and slides were stored at −80 °C (4, 24, 25).

### 2.3 Histochemistry and immunofluorescence (IF) staining

**FIGURE 1:**
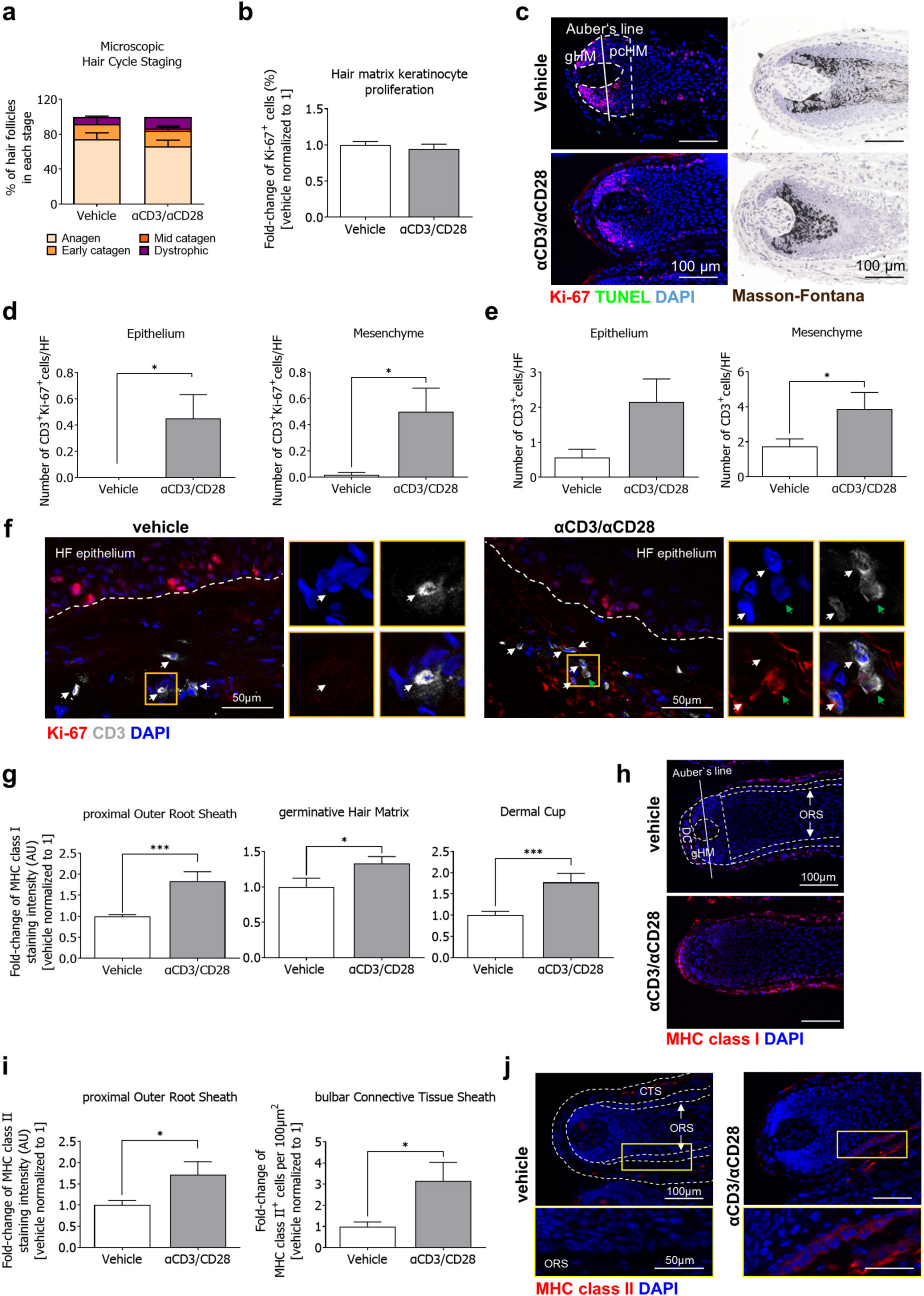
Stimulation with αCD3/αCD28 antibodies does not alter anagen/catagen conversion but leads to resident intra-/peri-follicular T-cell proliferation and bulb IP collapse *ex vivo*. HF were cultured in absence and presence of αCD3/αCD28 antibodies for 4 days. **(a)** Quantitative analysis of hair cycle staging (*n* = 4) and **(b)** hair matrix keratinocyte proliferation (Ki-67^+^ cells) within the gHM. *n* = 16–21 HFs/group from 4 donors. **(c)** Representative images of Ki-67/TUNEL immunostaining and Masson Fontana histochemistry. **(d,e)** Quantitative analysis of proliferating CD3^+^/Ki-67^+^ cell numbers **(d)** and CD3^+^ cells **(e)** in the HF epithelium and mesenchyme. Please note that inter-donor/experiments variations were detected. *n* = 29–30 HFs/group from 4 donors. **(f)** Representative images of CD3/Ki-67 immunostaining. White arrows: CD3^+^ cells, green arrows: CD3^+^/Ki-67^+^ cells. **(g)** Quantitative analysis of MHC class I expression in the pORS, gHM and DC. *n* = 14–15 HFs/group from 4 donors. **(h)** Representative images of MHC class I expression. **(i)** Quantitative analysis of MHC class II expression in the pORS, and MHC class II^+^ cell numbers in the bCTS. *n* = 15–16 HFs/group from 4 donors. **(j)** Representative images of MHC class II expression. Data are presented as mean ± SEM. Mann-Whitney U-test, *p < 0.05, ***p < 0.001. Scale bars = 100 μm. bCTS, bulbar connective tissue sheath; DC, dermal papilla; gHM, germinative hair matrix; HF, hair follicle; pORS, proximal outer root sheath.

Masson–Fontana staining for histochemical visualization of melanin was performed as described before (25–27) in *n* = 21 HFs/group from 3 donors (Supplementary Figure 1a) or *n* = 4–5 HFs/group from 1 donor (Supplementary Figure 1b). Ki-67 (1:800 in PBS; Cell Signaling Technology)/TUNEL (ApopTag^®^ Fluorescein *In Situ* Apoptosis Detection; Kit Merck Millipore) staining was performed as described in the manufacturer’s protocol and used to quantify proliferating and apoptotic keratinocytes in the hair matrix (25–27) in *n* = 16–21 HFs/group from 4 donors (Figure 1b) or *n* = 5 HFs/group from 1 donor (Supplementary Figure 1d). MHC class I and II staining was performed as previously described. In short, cryosections were fixed in acetone, and pre-incubated with 10% goat serum in TBS for MHCI or in PBS for MHCII. The sections were incubated with mouse anti-MHCI (M0738, clone W6/32, DAKO, 1:50) or mouse anti-MHCII (M0775, clone CR3/43, DAKO, 1:50) at 4 °C overnight and visualized with goat-anti-mouse-IgG-Rhodamine Red (111-095-144, Jackson ImmunoResearch, 1:200) for 45 min at RT (12, 13, 15, 28, 29). CD3 staining was performed as previously described. In short, cryosections were fixed in acetone, and pre-incubated with 5% BSA in PBS. The sections were incubated with mouse anti-CD3-AF647 (15-0038-42, clone UCHT1, Thermofisher, 1:50) at 4 °C overnight. Counterstaning with DAPI (1 μg/ml) was performed to visualize nuclei.

### 2.4 Quantitative (immuno-)histomorphometry

**FIGURE 2:**
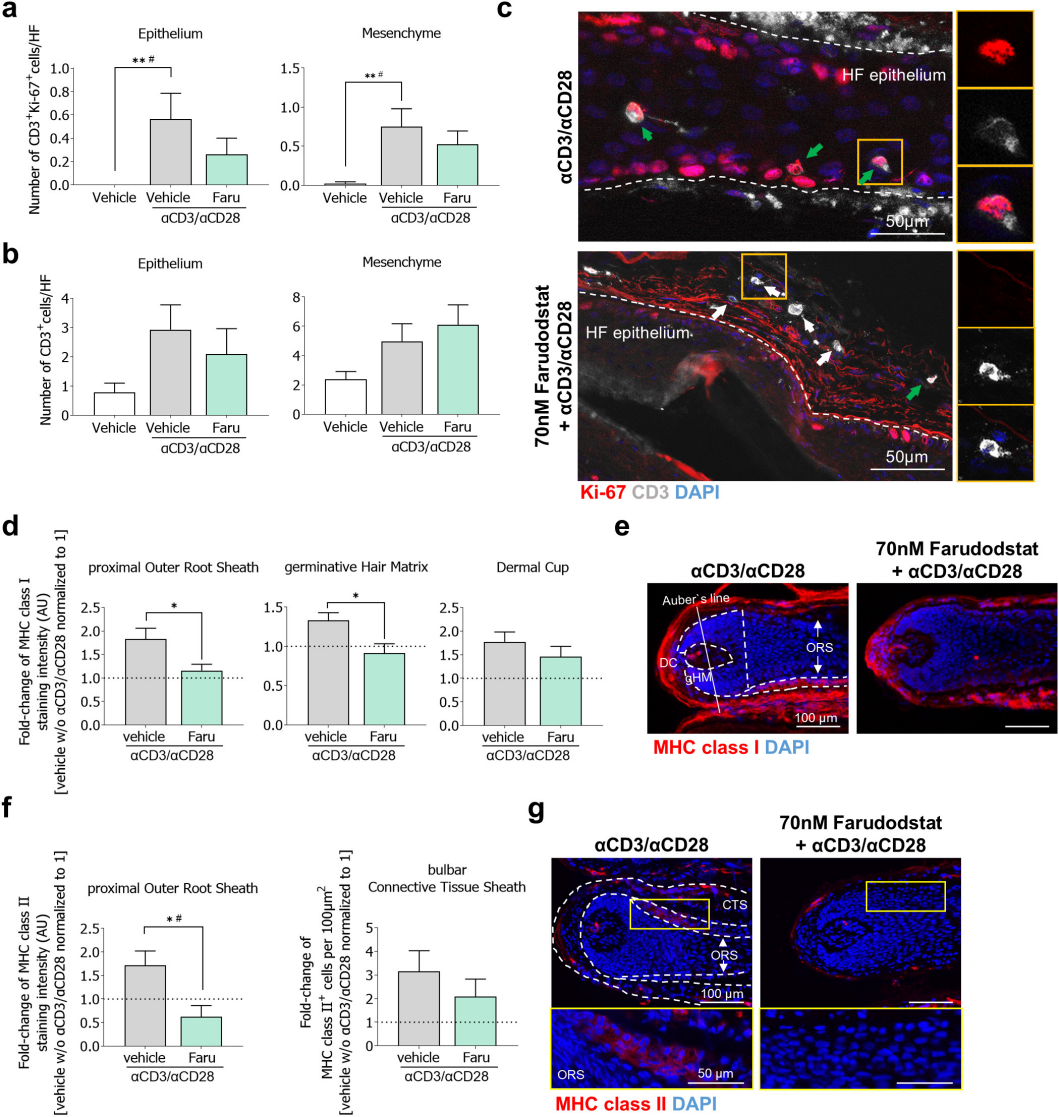
Farudodstat prevents αCD3/αCD28 antibody-induced resident T-cell proliferation and bulb IP collapse in human HFs *ex vivo*. HF were cultured with vehicle control or Farudodstat for 1 day and afterward αCD3/αCD28 antibodies were added for another 4 days. **(a,b)** Quantitative analysis of proliferating CD3^+^/Ki-67^+^ cell numbers **(a)** and CD3^+^ cells **(b)** in the HF epithelium and mesenchyme. *n* = 21–22 HFs/group from 3 donors. **(c)** Representative images of CD3/Ki-67 immunostaining. White arrows: CD3^+^ cells, green arrows: CD3^+^/Ki-67^+^ cells. **(d)** Quantitative analysis of MHC class I expression in the pORS, gHM and DC. *n* = 9–15 HFs/group from 3 independent donors. Data are normalized to vehicle control without αCD3/αCD28 stimulation (dotted line at 1). **(e)** Representative images of MHC class I expression. **(f)** Quantitative analysis of MHC class II expression in the pORS, and MHC class II^+^ cell numbers in the bCTS. *n* = 10–16 HFs/group from 3 independent donors. Data are normalized to vehicle control without αCD3/αCD28 stimulation (dotted line at 1). **(g)** Representative images of MHC class II expression. Data are presented as mean ± SEM. Kruskal Wallis with Dunn’s multiple comparison #*p* < 0.05, Mann-Whitney U-test, *p < 0.05, **p < 0.01. Scale bars = 100 μm. bCTS, bulbar connective tissue sheath; DC, dermal papilla; gHM, germinative hair matrix, HF, hair follicle; IP, immune privilege; pORS, proximal outer root sheath.

Images were taken using a Keyence fluorescence microscope (BZ9100 Keyence, Osaka, Japan) maintaining a constant set exposure time throughout imaging for further analysis. Staining immunoreactivity or the number of positive cells were counted in standardized reference areas with the software ImageJ (National Institutes of Health, Bethesda, MD, USA, open source). Hair cycle staging was performed utilizing Masson–Fontana staining and Ki-67/TUNEL immunofluorescence in *n* = 16–21 HFs/group from 4 donors (Figure 1a) or *n* = 5 HFs/group from 1 donor (Supplementary Figure 1c), according to established parameters (23, 30). MHC class I expression was measured in the dermal cup (DC), germinative hair matrix (gHM), and in the proximal outer root sheath (pORS) (11, 13, 15) of *n* = 14–15 HFs/group from 4 donors (Figure 1g), *n* = 9–15 HFs/group from 3 donors (Figure 2d) or *n* = 5 HFs/group from 1 donor (Supplementary Figure 1e), MHC class II expression was measured in the proximal ORS, and in the bulbar connective tissue sheath (bCTS) the number of positive cells per 100 μm^2^ were counted (11–13) in *n* = 15–16 HFs/group from 4 donors (Figure 1i) or *n* = 10–16 HFs/group from 3 donors (Figure 2f). The fluorescence staining intensity correlates with the target protein expression and was measured in the corresponding evaluation areas from each single HF and averaged within the experimental groups. The numbers of proliferating T-cells (CD3^+^/Ki-67^+^) cells were counted in epithelial and mesenchymal HF compartments of *n* = 29–30 HFs/group from 4 donors (Figures 1e,f) or *n* = 21–22 HFs/group from 3 donors (Figures 2a,b).

### 2.5 Statistical analysis

Statistical analyses were performed with GraphPad Prism9. Gaussian distribution was assessed with a D’Agostino & Pearson omnibus test. If data were normally distributed, either an ordinary one-way ANOVA with multiple comparison test to compare three or more groups, or a Student *t*-test to compare two groups, were performed. If data were not normally distributed, either a Kruskal-Wallis test with Dunn’s multiple comparison test to compare three or more groups, or a Mann-Whitney test to compare two groups, were used. Statistical tests are indicated in the figure legends. Data are expressed as mean ± SEM; *p* < 0.05*, *p* < 0.01**, *p* < 0.001***, were considered significant when two groups were compared, and *p* < 0.05#, *p* < 0.01##, *p* < 0.001###, were considered significant when three or more groups were compared.

## 3 Results

### 3.1 T cell receptor activation promotes resident T cell expansion and induces immune privilege collapse in healthy human hair follicles *ex vivo*

Under physiological conditions, T cells are relatively scarce within the epithelium and mesenchyme of healthy human HFs and locate mostly in the distal part of the HFs, outside of the IP protected areas, namely the bulge and the bulb (1, 31, 32). During full-length HF microdissection, a small number of T cells is retained, located within residual dermal tissue and within the intra- and peri-follicular regions of the HF (4). To investigate whether the activation of these HF resident T cells leads to bulbar IP collapse, we selectively activated their TCR using αCD3/αCD28 stimulation (6, 7) for 5 days *ex vivo*.

We initially confirmed that the selected experimental setting and dose of αCD3/αCD28 antibodies was not cytotoxic, confirmed by the absence of melanin clumping (Supplementary Figure 1a). Because obtaining sufficient anagen VI HFs after αCD3/αCD28 application is essential for accurately assessing IP status in the HF bulb, and given that our aim was to induce IP collapse rather than trigger premature catagen induction, we confirmed that the selected αCD3/αCD28 stimulation protocol did not alter the hair cycle or hair matrix keratinocyte proliferation *ex vivo* (Figures 1a–c). Despite detecting substantial inter-donor and inter-experiment variations, T cell proliferation was significantly higher in both the HF epithelium and mesenchyme following TCR engagement (Figures 1d,f). This led to an increased number of resident CD3^+^ cells, which reached statistical significance in the mesenchyme (Figures 1e,f), indicating successful resident T cell activation following TCR engagement with αCD3/αCD28.

Quantitative (immuno-)histomorphometry analysis of MHC class I and II expression, two key markers of IP collapse in human HFs (12, 15, 28, 29), revealed that TCR stimulation resulted in significantly enhanced ectopic expression of MHC class I in the pORS, gHM and DC (Figures 1g,h), and MHC class II expression in the pORS, as well as MHC class II^+^ cell numbers in the bulbar connective tissue sheath (bCTS) (Figures 1i,j).

These findings show that activation of T cells located within residual dermal tissue and HF resident intra- and peri-follicular T cells drives IP collapse in microdissected healthy human HFs *ex vivo*.

### 3.2 Farudodstat reduces resident T cell proliferation and prevents experimentally induced immune privilege collapse in healthy human hair follicles *ex vivo*

Dihydroorotate dehydrogenase plays a broad role in cell proliferation (33), and therefore its inhibition could also potentially affect hair matrix keratinocytes, cells essential for anagen maintenance and hair shaft production. Although preliminary data indicate that farudodstat selectively inhibits T cell proliferation, a potential advantage over other DHODH inhibitors (20), we first sought to ensure that our treatment would not interfere with the hair cycle. Therefore, in a proof-of-principle experiment, we initially verified that farudodstat treatment without αCD3/αCD28 stimulation did not cause cytotoxicity, affect the hair cycle, alter hair matrix keratinocyte proliferation or IP collapse marker expression in healthy human HFs *ex vivo* (Supplementary Figures 1c–e).

We next sought to investigate whether T cell proliferation plays a central role in the TCR activation–dependent collapse of the bulb IP. To test this, we co-administered the DHODH inhibitor farudodstat 1 day prior and during the stimulation with αCD3/αCD28 in HFs *ex vivo*. Short-term treatment with farudodstat tended to prevent the increase in CD3^+^ proliferation induced by αCD3/αCD28 stimulation in the HF epithelium and mesenchyme, but did not affect on CD3^+^ cell numbers (Figures 2a–c), most likely reflecting the need for a longer treatment period to impact overall cell counts. Additionally, the enhanced MHC class I expression resulting from TCR stimulation was significantly inhibited in the pORS and gHM when farudodstat was co-administered, while a modest, but non-significant reduction was observed in the DC (Figures 2d,e). Treatment with farudodstat significantly decreased the αCD3/αCD28-induced MHC class II expression in the pORS while MHC class II^+^ cell numbers in the bCTS were only minimally affected (Figures 2f,g).

Thus, while farudodstat only partially suppressed intra- and peri-follicular T cell proliferation *ex vivo*, it effectively prevented the induction of HF IP collapse triggered by TCR engagement.

## 4 Discussion

We demonstrate, for the first time, that engagement of the TCR on a small population of resident intra- and peri-follicular T cells in microdissected healthy human HFs is sufficient to induce HF IP collapse *ex vivo*, evidenced by elevated MHC class I and II expression. This effect is abrogated when T cell proliferation is blocked by DHODH inhibition using farudodstat. Our experimental approach provides a robust, cost-effective (relative to lesional AA scalp skin), and experimentally controllable model system that recapitulates an AA-like phenotype initiated specifically by resident T cell activation.

Several experimentally inducible models of AA have been successfully established, such as those employing IFNγ (11–15) or Substance P (16) treatment. While these models reliably reflect IP collapse and associated inflammatory damage, they do not recapitulate antigen-dependent TCR engagement, instead they primarily represent secondary downstream pathways of this process. Therefore, the availability of models that capture the initiation of AA at the stage of T cell activation is critical for advancing mechanistic understanding and facilitating the identification of novel therapeutic targets. This is particularly noteworthy in the context of the two proposed forms of AA: the antigen-independent type and the “classical” autoimmune-dependent form (1, 2). Furthermore, the study of co-stimulatory pathways in AA cannot be addressed in a purely cytokine-driven AA model. Indeed, addition of further T cell co-stimulatory molecules with relevance in AA, such as IL-2 or IL-15 (34, 35), to our model would be of interest to assess whether these can accelerate the effect of αCD3/αCD28 stimulation alone. Moreover, our model could be leveraged to selectively drive the expansion of specific T cell subtypes and to mechanistically investigate their contribution to AA pathogenesis, for example, the role of resident memory T cells in disease chronicity and relapse (36). Mimicking physiological T cell activation at the HF also allows to dissect earlier events of AA, for instance how HF-derived cytokines (e.g., IL-7, IL-15) (37, 38), stress signals, and local antigen presenting cells shape HF resident T cells before robust IFNγ production occurs. This is crucial for the identification of preventive or early interventional targets upstream of IFNγ and JAK signaling.

Although farudodstat was used as a proof-of-principle to confirm that T cell proliferation is a key upstream event leading to HF IP collapse, our data also supports its potential as a therapeutic candidate for AA. It should be noted, however, that farudodstat only partially reduced T cell proliferation, and had no effect on T cell numbers, possibly because it affects only specific T cell subsets or clones, which is known for other DHODH inhibitors (19, 21). The lack of an observable effect of short-term farudodstat treatment on T cell numbers may also be explained by the possibility that the reduced proliferation had not yet translated into a measurable decrease in total T cell counts. Once present following TCR stimulation, T cells may persist without undergoing immediate cell death. Furthermore, farudodstat may act primarily by reducing cytokine production, particularly IFNγ, thereby influencing downstream events such as the expression of IP collapse markers, while leaving overall T cell numbers largely unaffected. Indeed, despite the modest beneficial effect on αCD3/αCD28-induced T cell proliferation, farudodstat nevertheless efficiently prevented subsequent upregulation of MHC class I and II, supporting the concept that targeting T cell proliferation could represent an efficient therapeutic strategy in AA, potentially via blocking cytokine release. Previous work has demonstrated that the timing of DHODH inhibitor administration is critical in determining its effects on cytokine production. Specifically, selective DHODH inhibition with the small molecule inhibitor HOSU-53 during the initial activation of CD3^+^ T cells *in vitro* reduced both proliferation and IFNγ production. In contrast, DHODH inhibition after T cell activation suppressed proliferation and oxidative metabolism without substantially affecting cytokine output in this experimental setting (39).

Thus, further studies are warranted to assess whether prolonging farudodstat treatment would produce a more pronounced reduction in T cell proliferation and, consequently, T cell numbers. In addition, future experiments should compare preventive versus therapeutic administration to identify the optimal timing of farudodstat treatment. Such insights could provide valuable guidance also for potential clinical application. In parallel, quantifying the secretion of effector molecules such as IFNγ would help determine whether their reduction is a key factor mediating farudodstat’s beneficial effect in preventing IP collapse. Additionally, in this study we specifically aimed to induce key markers of IP collapse rather than changes in the hair cycle, as IP alterations precede hair cycle disruption in AA. However, it would be also interesting to determine whether increasing concentrations of αCD3/αCD28 can cause premature catagen induction, analogous to the dose-dependent effects of IFNγ, where low concentrations induce IP collapse and high concentrations trigger catagen induction (11, 40). Finally, preclinical studies assessing the efficacy of farudodstat in direct comparison with FDA-approved JAK inhibitors, such as baricitinib or ritlecitinib, which block cytokine signaling downstream of T-cell activation (41), are needed to determine whether inhibiting T-cell proliferation truly represents a viable therapeutic strategy for AA.

Taken together, our pilot data demonstrate that stimulation of HF resident T cells via TCR engagement with αCD3 and αCD28 antibodies can induce an AA–like phenotype in healthy human HFs *ex vivo*, characterized by T cell proliferation and IP collapse. This novel model for investigating AA pathogenesis can be used or further developed to evaluate candidate therapeutics targeting T cells and/or upstream events of IP collapse induction.

## Data Availability

The raw data supporting the conclusions of this article will be made available by the authors, without undue reservation.
