# Supplementary material for: Resident T cell activation leads to human hair follicle immune privilege loss ex vivo, which is prevented by the DHODH inhibitor farudodstat: relevance for alopecia areata
Source: Front Med (Lausanne). 2026 Apr 29;13:1810644. doi: 10.3389/fmed.2026.1810644 (PMC13169153; doi:10.3389/fmed.2026.1810644)
Supplement: Supplementary file 1 [file Image_1.pdf]

Supplementary Information

Resident T-cell activation leads to human hair follicle immune privilege loss ex vivo, which is prevented by the DHODH inhibitor farudodstat: relevance for alopecia areata

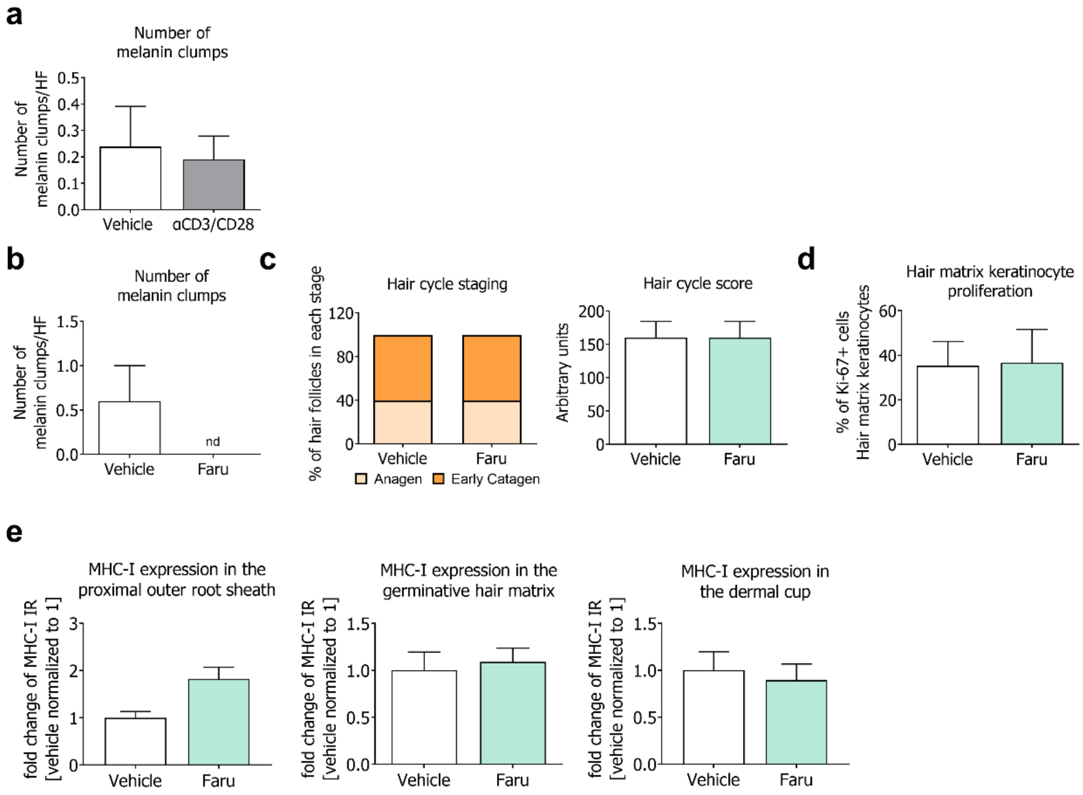

Supplementary Figure 1. Stimulation with αCD3/αCD28 is not cytotoxic, and Farudodstat treatment has no effect on the hair cycle, hair matrix keratinocyte proliferation, or IP marker expression. (a) HFs were cultured in absence and presence of αCD3/αCD28 antibodies for 4 days. Quantitative analysis of melanin clumping. n=21 HFs/group from 3 donors. (b-e) HFs were cultured with vehicle control or 70nM Farudodstat for 1 day and afterwards αCD3/αCD28 antibodies were added for another 4 days. (b) Quantitative analysis of melanin clumps (n = 4-5 HFs/group from 1 donor), (c) hair cycle staging and hair cycle score (n = 5 HFs/group from 1 donor), (d) hair matrix keratinocyte proliferation (Ki-67+ cells) within the gHM (n = 5 HFs/group from 1 donor). (e) Quantitative analysis of MHC class I expression in the pORS, gHM and DC (n = 5 HFs/group from 1 donors). Data are presented as mean ± SEM. Mann-Whitney U-test, *p<0.05. bCTS: bulbar connective tissue sheath, DC: dermal papilla, gHM: germinative hair matrix, HF: hair follicle, nd: not detected, pORS: proximal outer root sheath.
